# We need better evidence for social prescribing: call for action for better systems for collaboration and building evidence

**DOI:** 10.1177/17579139241294003

**Published:** 2025-07-05

**Authors:** N Howlett, K Brown, I Freethy, SW Mercer, G Özakıncı

**Affiliations:** Public Health and Applied Behaviour Change (PHAB) Lab, Department of Psychology, Sport and Geography, University of Hertfordshire, College Lane, Hatfield AL10 9AB, UK; Public Health and Applied Behaviour Change (PHAB) Lab, Department of Psychology, Sport and Geography, University of Hertfordshire, Hatfield, UK; Public Health and Applied Behaviour Change (PHAB) Lab, Department of Psychology, Sport and Geography, University of Hertfordshire, Hatfield, UK; Advanced Care Research Centre, Usher Institute, The University of Edinburgh, Edinburgh, UK; Division of Psychology, Faculty of Natural Sciences, University of Stirling, Stirling, UK

## Abstract

This opinion piece details the challenges associated with defining and evaluating social prescribing. It explores the types of data and considerations needed to produce a more robust evidence base in this area, concluding with a call to action for better collaboration and evidence building from a range of stakeholders.


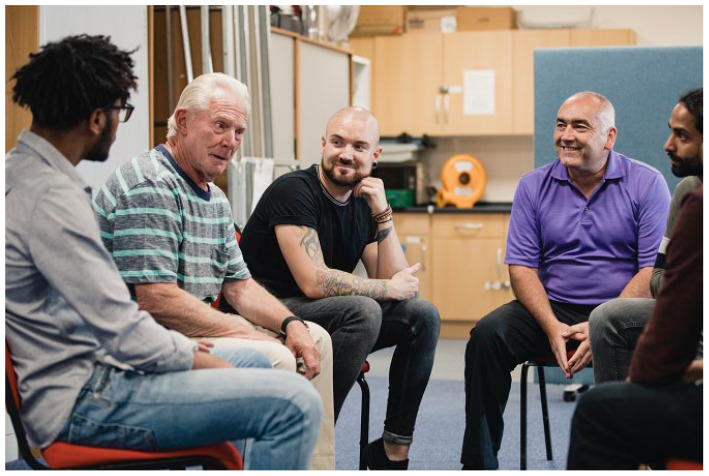


In 2019, Husk et al.^1^ stated ‘social prescribing is the topic of the moment’, and this remains the case. Globally, societies are struggling to find solutions to growing health inequalities and the impact of social determinants on creating (ill) health is increasingly recognised. Social prescribing is regarded as a way of mitigating health inequalities through better support of people in deprived areas^2^ and has become more important post-COVID-19.^3^

However, defining social prescribing and studying its implementation and effectiveness is not straightforward. It has been argued that ‘social prescribing is not a coherent thing, but rather an idea that has been interpreted and implemented in diverse ways’.^4^ Recently, Muhl et al.^5^ put forward an operational definition that made the useful distinction between the dual roles of social prescribers with ‘identification’ of non-medical needs (e.g. social isolation) and ‘connecting’ the person to community supports by co-producing a social prescription. The authors highlight that the identifier can be someone from community settings. However, in the United Kingdom, social prescribing is increasingly conducted by general practitioners (GPs) working in areas of high deprivation, through ‘link workers’ who take referrals from GPs and work as part of the primary care team.

## Current Evidence and Remaining Gaps

Given the interest among policy makers, voluntary sector, health and social care providers, and general public, to determine whether social prescribing services work, how, and for whom, it is critical to enable these services to grow in a rigorous and evidence-based way. A systematic review showed that despite positive impacts from social prescribing, neither success nor value for money can often be assessed adequately, due to studies with high risk of bias, variety in outcomes, and lack of control groups.^6^ Even when robust studies have been conducted, they often report no impact on health-related quality of life and mental health, with very little analysis of cost-effectiveness.^7^ A further review found a range of positive effects of social prescribing on mental health and wellbeing.^8^ However, there was still a lack of consistently robust outcomes or mixed-method evaluations. It has been stated that policy makers’ support for better evaluation of current social prescribing approaches is paramount before wider rollout.^7^ However, such an approach, termed ‘middle-ground research’ involving academic researchers, policymakers, National Health Service (NHS) staff, third sector organisations, and patients, is rare,^9^ and the evidence produced may not be acted upon. For example, evaluation of the Scottish Governments’ pilot study of link workers in GP practices did not produce convincing evidence of benefit,^10^ yet the national rollout proceeded anyway.

## What are the Barriers to Generating Quality Evidence for Social Prescribing?

Public health interventions are notoriously difficult to evaluate due to the complexity of the context and interacting systems involved. Husk et al.^1^ summarised the challenges of providing evidence for social prescribing into methodological (e.g. definitions and measurements), generalisability (e.g. relevance outside of a unique locality), and practical (e.g. limited capacity and expertise of local teams). Even a well-funded cluster randomised controlled trial in Scotland had problems fully implementing social prescribing in practice due to limited GP buy-in, a lack of collaborative leadership, poor team dynamics, limited link worker support, and competing innovations.^2^

These challenges also presented themselves in a recent mixed-methods evaluation of green health prescriptions (GHPr) in Scotland.^11^ The quantitative element attempted to monitor health and wellbeing outcomes, and health service utilisation at baseline and 12 weeks. The qualitative element involved interviewing referrers, link workers, activity providers, and service users. The interview findings showed that staff and service users found GHPrs acceptable, with reported improvements in a range of physical and mental health, and social outcomes for service users.^11^ However, the main barrier for staff, particularly referrers, was the lack of strong underpinning IT infrastructure to note that a referral had been made, communication with link workers, and feedback and data capture to reflect on service user progress.^11^ These problems prevented the quantitative element of the evaluation being possible.

If trackability is a core component of social prescribing efforts, the infrastructure for data capture needs to be easily implemented. The findings of a realist scoping review of methods of connecting primary care patients with community-based physical activity identified four methods of connection and 15 different processes.^12^ To be able to rigorously answer the questions of what works for whom and how and in what circumstances, systems need to be incorporated to understand all parts of the process of connection: approaches to identifying eligible and willing individuals; behaviour change strategies aiming to enhance the likelihood of undertaking the behaviour; and the method of connecting with community-based opportunity. A guidance on and indicators of robust evaluation and evidence synthesis concerning the connection process of social prescribing schemes already exists.^13^ However, this guidance relies on the availability of infrastructure to capture these data.

## Working Towards Better Evidence for Social Prescribing

In the UK, the NHS guide for social prescribing and community-based support^14^ recommends that the referral process should be easy, with routine codes in GP IT systems to record social prescribing referrals. The same guide outlines a Common Outcomes Framework (COF) for measuring the impact of social prescribing,^14^ which breaks down desired outcomes into impacts on the person (e.g. quality of life), health and care system (e.g. Accident & Emergency visits), and community groups (e.g. number of volunteers). The framework also advocates for routine monitoring data on referrals, service user demographics, contacts with link workers, and where a service user goes onto. In combination, collection of this data would enable a stronger evidence base on the positive outcomes and cost-savings that social prescribing might provide.

Routine data collection, linked to a COF, needs to be embedded in every social prescribing programme and the responsibility for this lies across all stakeholder groups. The recommendations in Box 1 will help achieve more robust evaluation and monitoring of social prescribing going forward.

**Box 1.** Recommendations for social prescribing evaluation and monitoring.

- Policy makers and commissioners need to make good quality data collection a requirement for funding future social prescribing approaches and provide additional resource so that frontline staff have the capacity for data collection and monitoring. - Frontline staff need to be trained in the basics of evaluation, consenting, and data collection, so that they feel comfortable to introduce this to service users and embed evaluation into their practice. - Improvements in data linkage in IT systems are needed so referrers can monitor the progress of service users. - There needs to be meaningful collaboration between academics, policymakers, and the services referring to, and providing social prescribing, in a ‘middle-ground’ approach to evidence production and implementation.
